# Gut Dysbiosis in the First-Passed Meconium Microbiomes of Korean Preterm Infants Compared to Full-Term Neonates

**DOI:** 10.3390/microorganisms12071271

**Published:** 2024-06-22

**Authors:** Sae Yun Kim, Young-Ah Youn

**Affiliations:** Department of Pediatrics, Seoul St. Mary’s Hospital, College of Medicine, The Catholic University of Korea, Seoul 06591, Republic of Korea; sysmile@catholic.ac.kr

**Keywords:** meconium, microbiome, dysbiosis, very preterm

## Abstract

Since gestational age (GA) is an important factor influencing the presence of specific microbiomes, we aimed to characterize the core microbiomes of preterm infants compared to full-term (FT) infants. This study investigated the differences in microbiota composition between very preterm (VP), moderate-to-late preterm (MLP), and FT neonates by examining the core microbiomes of a large cohort of Korean neonates. Meconium samples from 310 neonates with a GA range of 22–40 weeks were collected, and 16S rRNA analyses were performed; 97 samples were obtained from the FT, 59 from the VP, and 154 from the MLP group. Firmicutes, Bacteroidetes, and Proteobacteria were the phylum-level core microbiomes. Infants born before 37 weeks showed a disruption in the core microbiomes. At the phylum level, the relative abundance of Bacteroidetes was positively (*r* = 0.177, *p* = 0.002) correlated with GA, while that of Proteobacteria was negatively (*r* = −0.116, *p* = 0.040) correlated with GA. At the genus level, the relative abundances of *Bacteroides* and *Prevotella* were positively correlated with GA (*r* = 0.157, *p* = 0.006; *r* = 0.160, *p* = 0.005). The meconium of preterm infants exhibited significantly lower *α*-diversities than that of FT infants. *β*-diversities did not appear to differ between the groups. Overall, these findings underscore the importance of GA in shaping the early gut microbiome.

## 1. Introduction

The intestinal microbiota plays a critical role in the maturation and education of the host immune response, regulating the immune response, providing protection against the invasion of opportunistic pathogens, and regulating intestinal endocrine functions; it, therefore, has relevant health consequences [1,2,3]. Previous studies have suggested that the uterus and fetal meconium are sterile in healthy women. In other words, prior to birth, infants are isolated from exposure to environmental microorganisms [4], and microbes are acquired during and after birth; this phenomenon is referred to as the “sterile womb” hypothesis. In contrast, the “in utero colonization” hypothesis implies that the environment in utero is not sterile; therefore, the first-pass meconium may reflect the in utero microbial environment to a certain degree [5,6]. Regardless of whether meconium is colonized before, during, or after birth, the development and composition of the early gut microbiome have been shown to impact the well-being of infants [7,8].

Previously, perinatal events such as the mode of delivery have been shown to affect the types of microbes to which infants are exposed, resulting in the establishment of different patterns of gut microbiomes [9,10,11]. However, recently, it was reported that the mode of delivery had a transitional and small effect on neonates’ gut microbiota and that gestational age (GA) seems to be the main driver of neonates’ gut microbiota [12]. Because GA at birth represents the time of first microbial exposure, the microbiota composition of preterm infants is significantly different from that of full-term (FT) infants [13,14]. Very preterm (VP) infants present three main gut microbiota phyla: Firmicutes, Bacteroidetes, and Proteobacteria, which account for most community dynamics. Klopp et al. also demonstrated that the most abundant phyla in VP infants included Firmicutes, Bacteroidetes, Proteobacteria, and Actinobacteria in a multi-center cohort study in Germany [11]. Meanwhile, the preterm gut microbiome undergoes early disruption before achieving maturation and develops into lower biomass or diversity [11,15]. Due to the immature nature of the gut microbiota, preterm infants face vulnerability to dysbiosis, resulting in dysfunctional intestinal immune system processes [16].

Many studies have indicated that preterm morbidities are strongly associated with the gut microbiota. Stewart et al. reported that preterm babies who developed necrotizing enterocolitis (NEC) had lower diversity in their gut microbiota [17], and Olm et al. revealed an increased bacterial replication rate before NEC diagnosis, which leads to an imbalance in the concentrations of compounds in the gut environment and may subsequently trigger the onset of NEC [18]. Furthermore, Beghetti et al. suggested that early *Bifidobacterium* deficiency is related to adverse neurological outcomes in very low birth weight infants [19].

Since the meconium microbiome can reflect the underlying vulnerability of preterm infants, identifying distinctive compositions may help predict which premature infants are more vulnerable. Therefore, appropriate early-life intervention can be crucial for reducing complications and improving the survival rate of premature infants, possibly by predicting high-risk morbidities and hospital outcomes. The objective of this study was to assess differences in the microbiota composition of first-passed meconium between very preterm (VP), moderate-to-late preterm (MLP), and FT neonates. We aimed to characterize the core microbiomes of preterm infants at both the phylum and genus levels from a large cohort of Korean neonates.

## 2. Materials and Methods

### 2.1. Study Population and Sample Collection

This was a prospective cohort study performed at the Catholic University of Korea, Seoul St. Mary’s Hospital, which is a tertiary referral university hospital with a level IV neonatal intensive care unit (NICU). All neonates, including preterm and FT babies, who were admitted to the NICU between May 2021 and August 2023 were eligible for the study. The inclusion criteria were as follows: (1) first meconium stool sample was obtained within 72 h after birth during the study period, (2) admitted to the NICU regardless of the GA at birth, and (3) written informed consent was provided by the legal guardians. The exclusion criteria were as follows: (1) transferred to the NICU after being admitted to the nursery or another hospital, (2) first meconium stool passed 72 h or more after birth, and (3) for the FT group, infants who were primarily admitted for gastrointestinal problems.

All meconium samples were collected within 72 h of birth. Immediately upon passage, 1–2 mL of the infant’s first-pass meconium was collected from diapers using sterile spatulas, placed in sterile tubes, and stored at −20 °C. Within 48 h after collection, the specimens were transferred to a deep freezer set at −80 °C until DNA was extracted for microbiome analyses. The clinical course and progression of the infants included in the study were reviewed until death or discharge. The maternal and infant demographic and clinical data, including mode of delivery, GA, birth weight, and postnatal hospital outcomes, were obtained.

### 2.2. Fecal Microbiome Analysis Method

DNA was extracted from the meconium samples, and, for the target region (V3–V4), specific amplification PCR was conducted. Subsequently, dual index PCR was performed with an Illumina sequencing platform using the PCRBIO VeriFi Mix (PCR Biosystems^®^, London, UK) and Nextera ^®^ Index Kit V2 Set A (Illumina^®^, San Diego, CA, USA). After indexing, the final pooled library concentration and size were checked.

DNA was pooled and sequenced on an Illumina MiSeq platform, and the sequencing data of bacterial variability sites (V3–V4) were analyzed with a 16S metagenomics application. Then, the sequencing and data analysis were performed using Quantitative Insights into Microbial Ecology 2 (QIIME2; version 2020.2 and 2020.6) [20]. DNA reads less than 200 bp in length were omitted from the taxonomic analysis. Reads were then demultiplexed and denoised with DADA2 [21]. The denoised reads were trimmed at 15 bp and truncated at 260 bp, and chimeric reads were filtered out, resulting in a total of 3,994,640 processed reads ready for further analyses. The R package decontam (version 1.8.0) was used to filter out environmental contaminants from each sample type [22]. The detailed sampling, preprocessing, and analysis methods used for the meconium samples have been published elsewhere [13].

### 2.3. Definitions

Preterm birth was defined as any birth before 37 completed weeks of gestation. On the basis of GA, further subdivisions were made: neonates born before 32^0/7^ weeks of GA were referred to as the VP group, and neonates born between 32^0/7^ and 36^6/7^ weeks were referred to as the MLP group [23]. The FT group included infants who were born after 37 weeks of gestation.

Antenatal corticosteroid (ACS) was defined as at least one dose of ACS administered to the mother before delivery for fetal lung maturity. Premature rupture of membranes was defined as rupture before delivery if it lasted for >18 h. Histological chorioamnionitis was defined as described by Yoon et al. [24]. The GA at birth was calculated according to the last menstrual period or ultrasound during the first trimester. The bronchopulmonary dysplasia (BPD) definition was adopted from the National Institute of Child Health consensus on BPD severity [25]. Sepsis was determined based on positive blood culture results. Necrotizing enterocolitis (NEC) was defined as stage II or higher [26]. Feeding intolerance (FI) was defined as persistent gastric aspirates of >50% of the feed volume, with or without increased abdominal girth, in the absence of culture-positive sepsis or radiographic evidence of NEC for 48 h [27], more than 3 times per day, which did not allow the advancement of feeding > 10–20 mL/kg/day. Retinopathy of prematurity was defined as stage 3 or higher and/or requiring treatment [28,29].

### 2.4. Statistical Analyses

The clinical characteristics and microbiology data were compared among the VP, MLP, and FT groups. Continuous variables are summarized as the means with standard deviations, and categorical variables are presented as frequencies and percentages, as appropriate. We conducted one-way analysis of variance with the post hoc Bonferroni correction method. Alternatively, the Kruskal-Wallis test was performed if the normality of the variable was not confirmed. Differences in categorical variables were compared using the chi-square test for trend or Fisher’s exact test. Pearson correlation coefficients were calculated between GA and the relative abundance of bacterial taxa. The α-diversity was calculated using Shannon’s diversity index, and *β*-diversity was plotted using principal coordinate analysis of Bray–Curtis dissimilarity. All the statistical tests were 2-sided, and differences between the group correlations were considered significant at *p* < 0.05. All the statistical analyses were performed using SPSS, version 24 software (IBM Corp., Armonk, New York, USA).

## 3. Results

### 3.1. Clinical Characteristics of the Cohort Population

**Figure 1:**
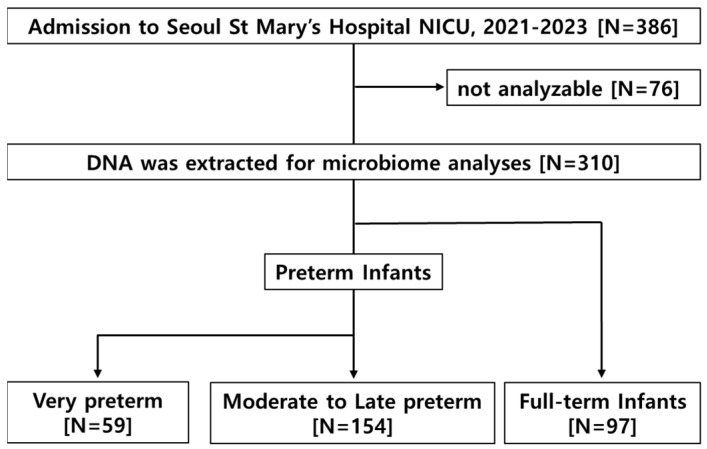
Flow chart of the study cohort. NICU, neonatal intensive care unit.

During the study period, the first-passed meconium was prospectively collected from 386 preterm and FT infants who were born between 22 weeks and 40 weeks of GA and admitted to the NICU of Seoul St. Mary’s Hospital. Despite careful handling, after preprocessing, 76 out of 386 meconium samples were not appropriate for analysis. A total of 310 infants were included in this study. Out of a total of 310 samples, 97 first-passed meconium samples were obtained from FT infants. Among the remaining 213 preterm infants, 59 infants born before 32^0/7^ weeks of GA composed the VP group and 154 infants born between 32^0/7^ and 36^6/7^ weeks of GA composed the MLP group (Figure 1).

Overall, 97 infants were delivered at term (mean GA 37.9 ± 1.0 weeks and mean birth weight 3128.1 ± 392.4 g), and 213 infants were delivered at < 37 weeks GA. Among the preterm babies, the mean GA and birth weight of the MLP group were 34.1 ± 1.3 weeks and 2241.4 ± 436.7 g, respectively, and the mean GA and birth weight of the VP group were 27.9 ± 2.5 weeks and 1096.0 ± 402.5 g, respectively. The proportion of ACS tended to decrease with increasing GA: VP (91.5%), MLP (72.7%), and FT (0.0%) groups (*p* for trend < 0.001). Prolonged antibiotic use (more than 3 days) in the antenatal period tended to decrease with increasing GA: VP (37.3%), MLP (14.9%), and FT (1.0%) groups (*p* for trend < 0.001), and the differences among the groups were all significant. The proportion of infants delivered by Cesarean section was significantly higher in the preterm groups, VP (94.9%) and MLP (94.8%), compared to the FT group (76.3%) (*p* = 0.002 and *p* < 0.001, respectively). The proportion of infants who developed NEC or FI tended to decrease with maturity: VP (39.0%), MLP (11.7%), and FT (5.2%) groups (*p* for trend < 0.001). Sepsis also showed a similar decreasing pattern according to GA: VP (27.1%), MLP (1.3%), and FT (0.0%) groups (*p* for trend < 0.001) (Table 1).

### 3.2. Comparison of Microbiomes According to Gestational Age

**Figure 2:**
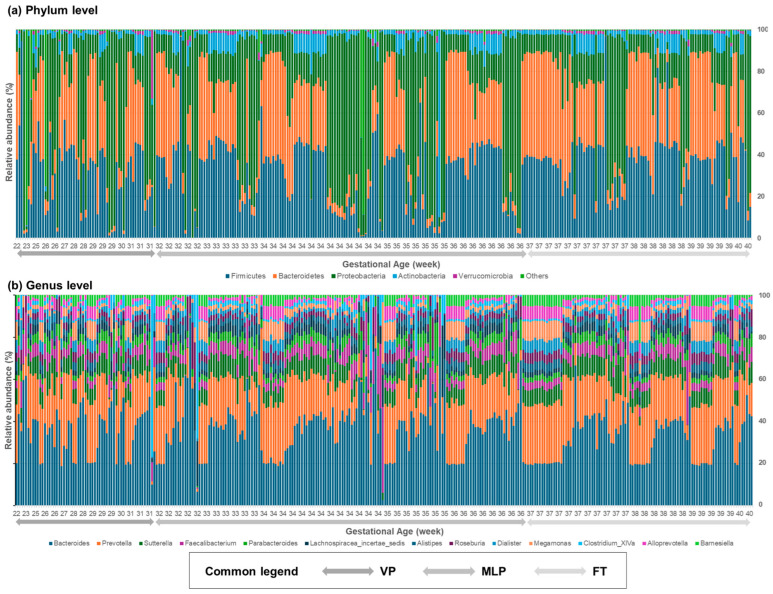
Relative abundances of bacterial taxa at the (**a**) phylum and (**b**) genus levels according to gestational age. Microbiomes of the first-passed stool samples obtained within 72 h after birth showed both phylum- and genus-level dysbiosis in neonates born before 37 weeks of gestation. At the phylum level, Firmicutes and Bacteroidetes were the most dominant phyla in the first meconium microbiome. At the genus level, *Prevotella* and *Bacteroides* were the most predominant species in the meconium samples. FT, full term; MLP, moderate-to-late preterm; VP, very preterm.

Compared to the first-passed meconium microbiomes of FT infants born after the 37th week of gestation, those of premature infants born before 37 weeks showed a disruption in the percentages of the core microbiomes at both the phylum and genus levels. However, comparisons between the VP and MLP groups, i.e., those born before and after 32 weeks of gestation, respectively, did not reveal differences. At the phylum level, Firmicutes and Bacteroidetes were dominant in the first-passed meconium microbiome (Figure 2a). At the genus level, *Prevotella* and *Bacteroides* were the most predominant species in the meconium samples (Figure 2b).

At the phylum level, Firmicutes, Bacteroidetes, and Proteobacteria were the core three distinctive microbiomes in the first-passed meconium samples, each accounting for approximately one-third of the total composition. Firmicutes accounted for 30.2% of the microbial community in the VP group, 30.5% of that in the MLP group, and 37.3% of that in the FT group (*p* = 0.003). The relative abundance of Bacteroidetes was 23.6% in the VP group, 24.7% in the MLP group, and 33.4% in the FT group (*p* < 0.001). The relative abundance of Proteobacteria was 37.7% in the VP group, 37.5% in the MLP group, and 24.4% in the FT group (*p* = 0.001). The next-most predominant phylum after the core three microbiomes was Actinobacteria, which comprised 3.5% of the microbial community in the VP group, 5.1% of that in the MLP group, and 4.3% of that in the FT group. However, among the above four phyla, the differences between the VP and MLP groups were not significant. Moreover, for the top three phyla, Firmicutes, Bacteroidetes, and Proteobacteria, the relative abundances between the MLP and FT groups were significantly different (*p* = 0.004, *p* = 0.001, and *p* = 0.002, respectively). Only the abundance of Bacteroidetes significantly differed between the VP and FT groups (*p* = 0.004).

**Figure 3:**
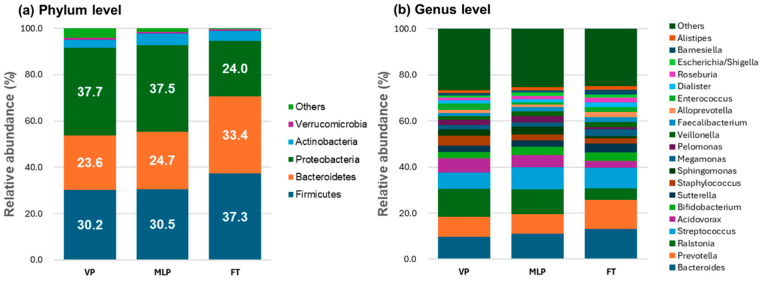
Meconium microbiome comparison between very preterm, moderate-to-late preterm, and full-term neonates. The bacterial composition of meconium was dominated by a few genera. Relative abundances and types of bacteria at the phylum level. The preterm group showed significantly lower abundances of Fermicutes and Bacteroidetes and a greater abundance of Proteobacteria (**a**). At the genus level, there were significant differences in the relative abundances of the 20 most dominant microbiota (**b**). The different colors correspond to different species names, and the length of the color block indicates the relative abundance of the species represented by the color block. The abscissa is the group name, and the ordinate is the relative abundance of the species.

Moreover, at the genus level, *Bacteroides*, *Prevotella*, and *Ralstonia* were predominant. The relative abundance of *Bacteroides* was 9.6% in the VP group, 11.0% in the MLP group, and 13.1% in the FT group (*p* = 0.006). The relative abundance of *Prevotella* was 8.8% in the VP group, 8.5% in the MLP group, and 12.7% in the FT group (*p* < 0.001). The relative abundance of *Ralstonia* was 12.2% in the VP group, 10.8% in the MLP group, and 5.0% in the FT group (*p* = 0.009). For the above three genera, the differences between the VP and MLP groups were not significant. However, the relative abundance of *Prevotella* in the FT group was significantly greater than that in the VP or MLP groups (*p* = 0.015 for VP vs. FT, and *p* < 0.001 for MLP vs. FT). The relative abundance of *Ralstonia* in the FT group was significantly lower than that in the VP or MLP groups (*p* = 0.027 for VP vs. FT, and *p* = 0.023 for MLP vs. FT). Among the remaining genera, eight were significantly different between the preterm group and the FT group: *Sutterella*, *Megamonas*, *Faecalibacterium*, *AlloPrevotella*, *Dialister*, *Roseburia*, *Barnesiella*, and *Alistipes*. Furthermore, the relative abundance of *Lactobacillus* was 0.402% in the VP group, 0.513% in the MLP group, and 0.461% in the FT group. The calculated ANOVA result do not include *p* values (Figure 3, Table A1 and Table A2).

### 3.3. Gestational Age and Microbiota Acquisition

**Figure 4:**
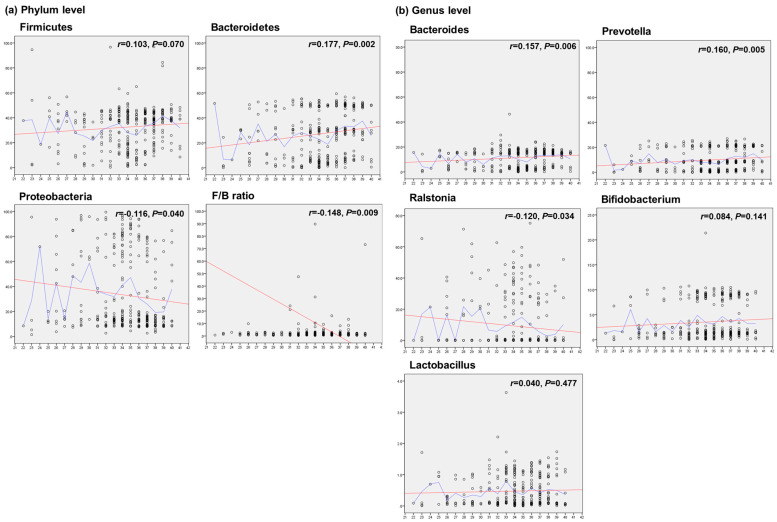
Scatter plots showing the correlation between GA and the relative abundances of the microbiota, which showed significant differences between groups at the phylum level (**a**) and at the genus level (**b**). At the phylum level, the relative abundances of Firmicutes and Bacteroidetes were positively correlated with GA. The relative abundance of Proteobacteria was negatively correlated with GA. The F/B ratio is calculated as the ratio between the two phyla Firmicutes and Bacteroidetes. At the genus level, the relative abundances of *Bacteroides* and *Prevotella* were positively correlated with GA, and the relative abundance of *Ralstonia* was negatively correlated with GA. The abundances of *Bifidobacterium* and *Lactobacillus* were not correlated with GA. Each point shows the median relative abundance for each gestational age. The red lines are the line of best fit at total through linear regression, and the blue lines are by the linear interpolation between elements in array. The abscissa is GA (week), and the ordinate is the relative abundance (%) of the species. The Pearson correlation coefficient was calculated. F/B ratio, Firmicutes to Bacteroidetes ratio; GA, gestational age.

To determine the effect of GA on the meconium microbiome, correlation analyses were conducted among the three combined groups to assess the relationships between GA and the relative abundances of several dominant phyla and genera. At the phylum level, the relative abundance of Bacteroidetes was positively correlated with GA (*r* = 0.177, *p* = 0.002), while the relative abundance of Proteobacteria was negatively correlated with GA (*r* = −0.116, *p* = 0.040). At the genus level, the relative abundances of *Bacteroides* and *Prevotella* were positively correlated with GA (*r* = 0.157, *p* = 0.006; and *r* = 0.160, *p* = 0.005, respectively). Conversely, the relative abundance of *Ralstonia* was negatively correlated with GA (*r* = −0.120, *p* = 0.034). Moreover, the abundances of *Bifidobacterium* and *Lactobacillus* were not correlated with GA. Additionally, the Firmicutes/Bacteroidetes (F/B) ratio was lower in infants with younger GA (Figure 4).

### 3.4. Microbial Diversity

**Figure 5:**
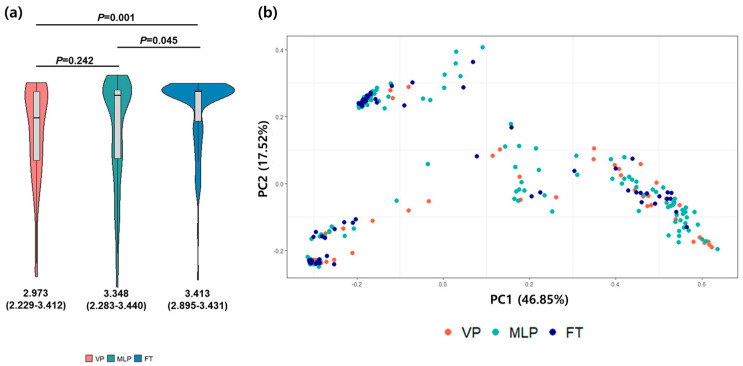
*α*-and *β*-diversities. Comparison of the α-diversities (Shannon Index) and *β*-diversities (Bray–Curtis dissimilarity) of preterm and full-term infants compared to those of full-term infants revealed that very preterm and moderate-to-late preterm infants had significantly lower *α*-diversities. The abscissa is the group name, the ordinate is the Shannon index of the species (**a**), and principal coordinate analysis (PCoA) reflects the distribution of the different samples (*R*^2^ = 0.027, *p* = 0.001). The dispersion of preterm infants and term infants was similar in the meconium (**b**). Each point represents one sample and is colored based on the group. FT, full-term; IQR, interquartile range; MLP, moderate-to-late preterm; VP, very preterm.

Compared to those of the FT group, the meconium of preterm infants exhibited significantly lower *α*-diversities. The median (IQR) Shannon index values were 2.973 (2.229–3.412) for the VP group, 3.348 (2.283–3.440) for the MLP group, and 3.413 (2.895–3.431) for the FT group. The differences between the preterm group and the FT group were significant (*p* = 0.001 for VP versus FT, *p* = 0.045 for MLP versus FT); however, the differences between the VP and MLP groups were not significant (*p* = 0.242). *β*-diversities did not appear to differ between the groups, indicating that the dispersions of meconium samples from preterm infants and term infants were similar (Figure 5).

## 4. Discussion

The neonatal microbiota starts to diversify quickly after birth. Compared to those of adults or older children, the infant microbiota is known to have lower diversity as well as an unstable and highly dynamic microbiota structure [30]. Our study revealed the microbiome composition of the first-passed meconium in Korean preterm and FT neonates, offering valuable insights into the impact of GA on gut dysbiosis in newborns. First, Firmicutes, Bacteroidetes, and Proteobacteria constituted the core microbiome. As the GA increased, there was a significant increase in the relative abundance of Bacteroidetes and a decrease in that of Proteobacteria, and the combined relative abundance of Firmicutes and Bacteroidetes increased in the following order: FT, MLP, VP. Second, we found that the microbiomes of the meconium of preterm infants exhibited lower *α*-diversities and significant disruptions in the core microbiome composition at both the phylum and genus levels compared to those of FT infants. This highlights a notable difference in the perinatal gut microbiome between preterm and full-term infants. Third, our analysis revealed statistically significant differences in the meconium microbiome composition between preterm and FT infants (VP versus FT and MLP versus FT), whereas evidence of differences between VP and MLP infants was scarce. Overall, these findings underscore the importance of GA in shaping the early gut microbiome of newborns.

Modeling the gut microbiota in neonates suggests competition between three phyla, namely, Firmicutes, Bacteroidetes, and Proteobacteria, which explained most of the observed community dynamics. Recently, a multicenter cohort study in Germany showed that GA at birth was significantly associated with the composition of meconium from VP infants, and the most abundant phyla included Firmicutes, Bacteroidetes, Proteobacteria, and Actinobacteria [11]. We also observed that Firmicutes, Bacteroidetes, and Proteobacteria constituted the majority of the microbiota in the VP group and MLP group, and the combined relative abundances of Firmicutes and Bacteroidetes were 53.8% in the VP group, 55.2% in the MLP group, and 70.7% in the FT group. Considering that Firmicutes and Bacteroidetes constitute more than 90% of the gut microbiota in healthy adults [31], these findings indicated that the meconium microbiota of FT infants is more similar to that of adults than to that of VP or MLP infants. In other words, the gut microbiomes of VP or MLP infants are immature and initially disrupted.

Furthermore, Rinninella et al. reported that healthy guts have bacteria of the phylum Bacteriodetes [32], and Shin et al. reported that the healthy gut of humans has relatively low abundance of the phylum Proteobacteria, which indicates that there are correlations between an increase in the abundance of Proteobacteria and disease states [33]. These findings are consistent with our finding that, as GA increased, a significant increase in the relative abundance of Bacteroidetes and a decrease in the relative abundance of Proteobacteria were observed.

Additionally, we observed a greater F/B ratio in earlier GA neonates. The F/B ratio has an important influence on regulating host energy metabolism, and an elevated F/B ratio indicates that effective energy intake is mediated by the gut microbiota [34]. Based on previous findings [35], it could be suggested that a higher F/B ratio in earlier GA neonates reflects increased fetal energy requirements for growth and development. Furthermore, our study revealed that the relative abundances of the core microbiomes—Bacteroidetes and Proteobacteria at the phylum level and *Bacteroides*, *Prevotella*, and *Ralstonia* at the genus level—are correlated with GA. These findings support the notion that GA is one of the most important factors influencing gut microbiota colonization.

Similarly, Aguilar-Lopez et al. emphasized that GA is an important factor that influences the gut microbiota in infants [36]. From birth, the immune system is predisposed to distinguish and destroy invading microbes, and, in this context, the human microbiota plays a vital role in preventing the growth of pathogens and modulating immune pathways. The interruption of the intrauterine environment in the third trimester results in an immature immune and intestinal system, which results in the inappropriate development of the gut microbiota [37].

Some previous studies have investigated the origin of the fetal gut microbiome. The vaginal microbiota is different from the intestinal microbiota, and they are dominated by *Lactobacillus* species, which produce lactic acid, maintain a low vaginal pH, and prevent dysbiosis and infection that can reach the fetus [38]. In a recent study, the relative abundance of *Lactobacillus* in preterm neonates was greater than that in term neonates [39]. However, our study revealed that *Lactobacillus* occupied a small part of the whole meconium microbiome, and the differences in the relative abundance of each group did not reach statistical significance: 0.402% in the VP group, 0.513% in the MLP group, and 0.461% in the FT group. This could be attributed to our cohort primarily consisting of infants delivered via Cesarean section (89.0%, N = 276), which may diminish the impact of vertical bacterial transmission of *Lactobacillus* from the maternal vaginal canal to the fetus.

From the current analyses, we found that many genera in the meconium microbiome of newborns were similar to those identified in the microbiota of maternal amniotic fluid, as pointed out by Ardissone et al. [39]: *Bacteroides*, *Prevotella*, *Streptococcus*, *Bifidobacterium*, *Staphylococcus*, *Enterococcus*, *Escherichia*, and *Lactobacillus*. One possible explanation is that the fetus starts to swallow a large amount of amniotic fluid during the last trimester of pregnancy (> 28 weeks) [40].

There were significant differences observed in the relative abundances of the core microbiota and *α*-diversities between the preterm group and the FT group. However, it was challenging to discern distinctly notable differences between the VP and MLP groups, suggesting that these two preterm subgroups may exhibit similar degrees of immaturity. Considering mother-to-infant bacterial transmission, two mechanisms have been proposed. During the 24th–28th weeks of gestation, the vaginal microbiota is primarily transmitted to the placenta and/or amniotic fluid through ascending migration from the vagina. Later, during the third trimester (after the 28th week of gestation), maternal gut bacteria are predominantly transmitted to the fetal intestine, which affect the meconium microbiota [41]. However, because we compared the VP group (<32 weeks of gestation) with the MLP group (≥32 and <37 weeks of gestation), the aforementioned two mechanisms could have occurred in the VP group, which might have affected the statistical significance.

Overall, distinct differences can be observed in the intestinal microbiota composition and structure between preterm and FT infants in their first stool following birth. Whether this difference in intestinal microbiota is related to differences in prognosis remains unclear. Since microbiota development is associated with infant gut maturity, especially in very preterm infants, there is a risk of delayed microbiota development, which may necessitate therapeutic intervention. The primary strength of our study lies in being the first to explore the meconium microbiome of Korean preterm and FT neonates, shedding light on gut dysbiosis in preterm neonates. Additionally, our study involves the largest prospective cohort of neonates in Korea to date, ensuring a representative sample across racial demographics. There are several limitations to this study. First, the inclusion of full-term neonates admitted to the NICU may not adequately represent a healthy control group. Second, the study spanned two years, leading to the microbiome analysis processes, such as DNA extraction and amplification, being conducted at different times, which might have led to intraclass variability. We hope that our findings will lay the groundwork for understanding an aspect of the human microbiome and will contribute to the design of future studies that focus on the meconium microbiome.

## 5. Conclusions

In summary, compared with those of FT infants, the microbiomes of the meconium of preterm infants exhibited lower *α*-diversities and significant disruptions in the core microbiome composition at both the phylum and genus levels. An increasing abundance of Bacteroidetes and a decreasing abundance of Proteobacteria were observed as GA increased. However, no significant differences in the meconium microbiome composition were observed between VP and MLP infants. Overall, these findings underscore the importance of GA in shaping the early gut microbiome of newborns and fuel the need to understand the development of the gut microbiota and for earlier therapeutic and preventative strategies for vulnerable preterm infants in future studies.

## Figures and Tables

**Table 1:** Maternal and neonatal characteristics of the study population.

	VP (<32 wks) [N = 59]	MLP (≥32 wks and < 37 wks) [N = 154]	FT (≥37 wks) [N = 97]	*p*	p*		
					VP vs. MLP	VP vs. FT	MLP vs. FT
Assisted pregnancy	10 (16.9%)	40 (26.0%)	6 (6.19%)	0.022	0.207	0.054	0.000
Antenatal corticosteroid use	54 (91.5%)	112 (72.7%)	0 (0.0%)	<0.001	0.003	<0.001	<0.001
PROM > 18 h	14 (23.7%)	19 (12.3%)	5 (5.2%)	0.001	0.056	0.001	0.077
HCAM	9 (15.3%)	1 (0.6%)	0 (0.0%)	<0.001	<0.001	<0.001	>0.999
Maternal antibiotics > 3 d	22 (37.3%)	23 (14.9%)	1 (1.0%)	<0.001	0.001	<0.001	<0.001
Maternal DM	10 (16.9%)	20 (13.0%)	9 (9.3%)	0.157	0.510	0.207	0.423
Oligohydramnios	14 (23.7%)	7 (4.5%)	1 (1.03%)	<0.001	<0.001	<0.001	0.045
Placenta abruption	7 (11.9%)	3 (1.9%)	1 (1.03%)	0.001	0.005	0.005	>0.999
Maternal preeclampsia	17 (28.8%)	35 (22.7%)	0 (0.0%)	<0.001	0.376	<0.001	<0.001
Cesarean section	56 (94.9%)	146 (94.8%)	74 (76.3%)	<0.001	>0.999	0.002	<0.001
Gestational age ^a^, week	27.9 ± 2.5	34.1 ± 1.3	37.9 ± 1.0	<0.001	<0.001	<0.001	<0.001
Birth weight ^a^, g	1096.0 ± 402.5	2241.4 ± 436.7	3128.1 ± 392.4	<0.001	<0.001	<0.001	<0.001
Male	34 (57.6%)	87 (56.5%)	56 (57.7%)	0.977	>0.999	>0.999	0.896
Requiring intubation at birth	32 (54.2%)	7(4.5%)	3 (3.1%)	<0.001	<0.001	<0.001	0.745
RDS	49 (83.1%)	41 (26.6%)	13 (13.4%)	<0.001	<0.001	<0.001	0.017
NEC or FI	23 (39.0%)	18 (11.7%)	5 (5.2%)	<0.001	<0.001	<0.001	0.115
BPD	23 (39.0%)	0 (0.0%)	0 (0.0%)	<0.001	<0.001	<0.001	-
Sepsis	16 (27.1%)	2 (1.3%)	0 (0.0%)	<0.001	<0.001	<0.001	0.524
ROP requiring surgery	9 (15.3%)	0 (0.0%)	0 (0.0%)	<0.001	<0.001	<0.001	-
Weight at discharge ^a^, g	3435.5 ± 910.2	2587.1 ± 412.5	3244.0 ± 501.5	<0.001	<0.001	0.124	<0.001

Values are presented as mean with standard deviation for continuous variables or frequencies with percentages for categorical variables, as appropriate. *p* values were calculated with the χ^2^ test for trends in categorical variables and one-way ANOVA for continuous variables between preterm and full-term infants. The p* values were compared between the VP and MLP groups, between the VP and FT groups, and between the MLP and FT groups through Fisher’s exact test (categorical variables), post hoc Bonferroni correction, or one-way ANOVA (continuous variables ^a^). BPD, bronchopulmonary dysplasia, FI, feeding intolerance; FT, full term; HCAM, historical chorioamnionitis; MLP, moderate-to-late preterm; NEC, necrotizing enterocolitis; PROM, premature rupture of membrane; RDS, respiratory distress syndrome; VP, very preterm.

## Data Availability

The datasets analyzed during the current study are not publicly available. The information contained in the data must be protected as confidential and will only become available to those individuals who have obtained permission from the data review board and the IRB of Seoul St. Mary’s Hospital to access and use these data for permitted research activities. The original contributions presented in the study are included in the article materials, and further inquiries can be directed to the corresponding author.

## Appendix A

**Table A1:** The average Relative abundance in Phylum level, at meconium of preterm and term neonates.

Phylum	Relative Abundance (%)			*p*	*p**		
	VP [N = 59]	MLP [N = 154]	FT [N = 97]		VP vs. MLP	VP vs. FT	MLP vs. FT
Firmicutes	30.2	30.5	37.3	0.003	>0.999	0.026	0.004
Bacteroidetes	23.6	24.7	33.4	<0.001	>0.999	0.004	0.001
Proteobacteria	37.7	37.5	24.0	0.001	>0.999	0.017	0.002
Actinobacteria	3.5	5.1	4.3	0.190	0.234	>0.999	0.929
Verrucomicrobia	1.0	0.7	0.6	0.389	0.750	0.554	>0.999
Others	4.0	1.5	0.5				

*p* value was calculated through one-way ANOVA test and *p** value was comparing VP vs. MLP, VP vs. FT, and MLP vs. FT through post-hoc Bonferroni test of one-way ANOVA. FT, full-term infants; MLP, moderate to late preterm infants; VP, very preterm infants.

**Table A2:** The average Relative abundance in Genuslevel, at meconium of preterm and term neonates.

Genus	Relative Abundance (%)			*p*	*p**		
	VP [N = 59]	MLP [N = 154]	FT [N = 97]		VP vs. MLP	VP vs. FT	MLP vs. FT
*Bacteroides*	9.6	11.0	13.1	0.006	0.532	0.007	0.065
*Prevotella*	8.8	8.5	12.7	<0.001	>0.999	0.015	<0.001
*Ralstonia*	12.2	10.8	5.0	0.009	>0.999	0.027	0.023
*Streptococcus*	7.0	9.5	8.9	0.285	0.341	0.806	>0.999
*Acidovorax*	6.2	5.3	3.0	0.070	>0.999	0.110	0.187
*Bifidobacterium*	2.9	3.8	3.6	0.275	0.329	0.708	>0.999
*Sutterella*	2.6	2.8	3.8	<0.001	>0.999	0.002	<0.001
*Staphylococcus*	4.2	2.5	2.3	0.374	0.617	0.584	>0.999
*Sphingomonas*	2.8	3.5	0.9	0.153	>0.999	0.810	0.162
*Megamonas*	1.9	1.8	3.1	<0.001	>0.999	0.023	0.001
*Pelomonas*	2.2	2.7	1.0	0.061	>0.999	0.540	0.056
*Veillonella*	1.6	2.1	2.1	0.258	0.358	0.453	>0.999
*Faecalibacterium*	1.5	1.8	2.3	0.001	0.375	0.001	0.013
*Alloprevotella*	1.4	1.3	2.2	0.001	>0.999	0.032	0.001
*Enterococcus*	2.6	0.9	2.0	0.260	0.410	>0.999	0.764
*Dialister*	1.3	1.3	2.1	<0.001	>0.999	0.013	<0.001
*Roseburia*	1.3	1.3	2.1	<0.001	>0.999	0.004	<0.001
*Escherichia/Shigella*	0.8	1.6	1.4	0.690	>0.999	>0.999	>0.999
*Barnesiella*	1.2	1.1	2.0	<0.001	>0.999	0.022	<0.001
*Alistipes*	1.2	1.2	1.7	0.001	>0.999	0.015	0.002
Others	26.7	25.4	24.7				
*Lactobacillus*	0.402	0.513	0.461	0.340	0.461	>0.999	>0.999

*Lactobacillus* is included in Others. FT, full-term infants; MLP, moderate to late preterm infants; VP, very preterm infants. *p* value was calculated through one-way ANOVA test and *p** value was comparing VP vs. MLP, VP vs. FT, and MLP vs. FT through post-hoc Bonferroni test of one-way ANOVA.
